# Prolonged action potential duration and dynamic transmural action potential duration heterogeneity underlie vulnerability to ventricular tachycardia in patients undergoing ventricular tachycardia ablation

**DOI:** 10.1093/europace/euy260

**Published:** 2018-11-29

**Authors:** Neil T Srinivasan, Michele Orini, Rui Providencia, Mehul B Dhinoja, Martin D Lowe, Syed Y Ahsan, Anthony W Chow, Ross J Hunter, Richard J Schilling, Peter Taggart, Pier D Lambiase

**Affiliations:** 1 Department of Cardiac Electrophysiology, The Barts Heart Center, St Bartholomew’s Hospital, West Smithfield, London, UK; 2 Institute of Cardiovascular Science, University College London, London, UK

**Keywords:** Ventricular tachycardia, Dispersion of repolarization, Transmural dispersion of repolarization, Action potential duration

## Abstract

**Aims:**

Differences of action potential duration (APD) in regions of myocardial scar and their borderzones are poorly defined in the intact human heart. Heterogeneities in APD may play an important role in the generation of ventricular tachycardia (VT) by creating regions of functional block. We aimed to investigate the transmural and planar differences of APD in patients admitted for VT ablation.

**Methods and results:**

Six patients (median age 53 years, five male); (median ejection fraction 35%), were studied. Endocardial (Endo) and epicardial (Epi) 3D electroanatomic mapping was performed. A bipolar voltage of <0.5 mV was defined as dense scar, 0.5–1.5 mV as scar borderzone, and >1.5 mV as normal. Decapolar catheters were positioned transmurally across the scar borderzone to assess differences of APD and repolarization time (RT) during restitution pacing from Endo and Epi. Epi APD was 173 ms in normal tissue vs. 187 ms at scar borderzone and 210 ms in dense scar (*P* < 0.001). Endocardial APD was 210 ms in normal tissue vs. 222 ms in the scar borderzone and 238 ms in dense scar (*P* < 0.01). This resulted in significant transmural RT dispersion (ΔRT 22 ms across dense transmural scar vs. 5 ms in normal transmural tissue, *P* < 0.001), dependent on the scar characteristics in the Endo and Epi, and the pacing site.

**Conclusion:**

Areas of myocardial scar have prolonged APD compared with normal tissue. Heterogeneity of regional transmural and planar APD result in localized dispersion of repolarization, which may play an important role in initiating VT.

**What’s new?**

- This is the first human study to examine dynamic transmural repolarization changes in scar substrate of ventricular tachycardia ablation patients.
- Areas of myocardial scar, regardless of pathology, have prolonged repolarization compared with normal voltage tissue.
- The presence of scar decreases the transmural repolarization gradient, the extent of which is dependent on the endocardial and epicardial scar characteristics.
- Alterations in transmural activation recovery interval gradient result in localized transmural dispersion of repolarization, dependent on the scar pattern and the activation sequence, which may play an important role in arrhythmogenesis.

## Introduction

Ventricular tachycardia (VT) is an important cause of defibrillator therapy and cardiac death. The arrhythmic substrate may be a region of enhanced automaticity, but is more frequently related to a region of fibrosis that facilitates re-entry.

Myocardial scar is most commonly associated with myocardial infarction or cardiomyopathic processes. Within regions of scar, areas of surviving tissue serve as sites of slow conduction and conduction block creating the necessary conditions for re-entry. Additionally, the scar borderzone is an important substrate for VT, with several studies^1^^,^^2^ demonstrating the efficacy of catheter ablation of this region. It is likely that the VT wavefront is heterogeneous, involving epicardial (Epi) loops^2^ or isthmuses, as well as taking a complex 3D path involving the endocardium, mid-myocardium, and the epicardium.^3^

However, the fundamental regional and transmural electrophysiological properties of action potential duration (APD), and repolarization time (RT) across scar and scar borderzone are poorly defined in the intact human heart. Repolarization time dispersion plays an important role in initiating and sustaining arrhythmia, but its heterogeneity across scar and scar borzerzone is unknown. *In vitro* studies in failing human hearts have shown prolonged APD and reduced transmural APD gradients,^4^ while peri-operative surgical mapping studies^5^ have demonstrated a lack of transmural APD gradients during ischaemia. These properties have not been dynamically studied in the intact human heart in conditions of chronic scar that are capable of sustaining VT. We aimed to investigate the planar and transmural properties of APD and RT across the borderzone of scar and normal tissue in patients undergoing VT ablation. This has important implications especially when attempting to construct clinically relevant computational models or design novel therapeutic interventions.

## Methods

### Clinical electrophysiological study

Endocardial (Endo) access to the left ventricle (LV) was obtained via the retrograde and transseptal approaches in all patients. Epicardial mapping was performed in all patients, as part of the clinical ablation study. Pericardial puncture was performed using the subxiphoid approach. Substrate maps were created during sinus rhythm, using CARTO 3D mapping system (Biosense Webster, Inc., Diamond Bar, CA, USA). Mapping was performed using a PentaRay (PentaRay, Biosense Webster, Inc.) or DecaNav mapping catheter (DecaNav, Biosense Webster, Inc.) with impedance parameters scaled to ensure tissue contact. Normal myocardium was defined as tissue with a bipolar voltage >1.5 mV, dense scar was defined as a bipolar voltage <0.5 mV, and scar borderzone was defined as a bipolar voltage 0.5–1.5 mV, consistent with previously published data.^1^^,^^2^ Though debate exists regarding the optimal voltage cut-off to define Epi scar^6^^,^^7^ we sought to use a consistent value for both epicardium and endocardium as previously published,^8^ in order to provide consistency to the APD measurements. Following this, activation mapping was performed if haemodynamically tolerated. Finally areas of late potentials and mid-diastolic potentials were identified.

### Research protocol

**Figure 1:**
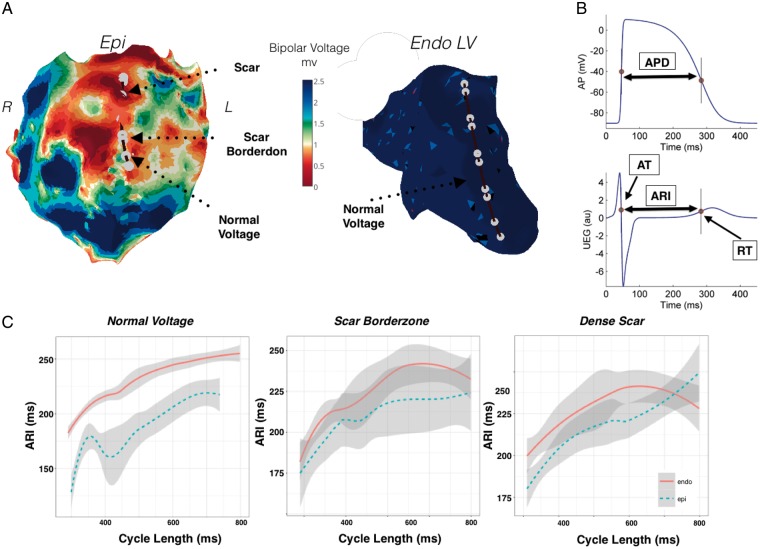
Restitution studies in a patient with previous myocarditis and predominant epicardial scar (Patient 1). (*A*) Endocardial and epicardial bipolar voltage maps highlighting a large region of anterior epicardial scar. Position of catheters simultaneously placed endocardially and epicardially for transmural recording is shown. (*B*) The Wyatt method was used to analyse RT. AT measured as d*V*/d*t*_min_ of the QRS and RT as the d*V*/d*t*_max_ of the unipolar intracardiac electrogram T-wave. ARI (ARI = RT − AT), was measured as a surrogate marker of APD. (*C*) ARI restitution during endocardial pacing, across epicardial normal voltage tissue, epicardial scar borderzone, and epicardial dense scar. APD, action potential duration; ARI, activation recovery interval; AT, activation time; Endo, endocardium; Epi, epicardium; NS, non-significant; RT, repolarization time.

Decapolar catheters, DecaNav (DecaNav, Biosense Webster, Inc.) were placed epicardially and endocardially and aligned to record across a geometrically opposed transmural area, traversing healthy tissue, scar-borderzone, dense scar, or all three (*Figure 1*). S_1_–S_2_ restitution curves were then performed at twice the diastolic capture threshold before clinical ablation, from the endocardium and epicardium in these catheter positions, as previously described.^9^ In each region, steady state was achieved by pacing at basic cycle length of 600 ms for 3 min. Following this an S_1_–S_2_ protocol was performed beginning with an extra stimulus (S_2_) at 1000 ms. The S_1_–S_2_ coupling interval was then decremented in 50 ms steps until an S_2_ of 400 ms, then by 20 ms intervals between 400 and 300 ms, and thereafter, in 5 ms steps until effective refractory period (ERP) of the tissue. At ERP an S_2_ stimulus at 10 ms + ERP was applied followed by further decrementing S_2_ in steps of 2 ms to confirm ERP. All patients gave informed consent, the study was approved by our regional ethics board (LO10/H0715/19) and complied with the declaration of Helsinki.

### Data analysis

Data analysis methodology has been previously described elsewhere by our group.^9^ Briefly, unipolar electrograms filtered at 0.05–500 Hz were recorded at a sampling rate of 2000 Hz (Bard Clearsign, CR Bard, NJ, USA). Local activation time (AT) was calculated as the interval from pacing stimulus to the minimum of the first derivative of the unipolar QRS complex (d*V*/d*t*_min_).^10^ Local RT was defined using the Wyatt method, as the maximum the first derivative of the unipolar T-wave (d*V*/d*t*_max_).^10^ Activation recovery interval (ARI) an accepted surrogate marker of APD and was calculated as ARI = RT − AT^10^ (*Figure 1B*). For unipolar recording a reference catheter was placed in the inferior vena cava to minimize far field interference on the signal. Data analysis was conducted using a MATLAB GUI that allows one to review and correct activation/repolarization markers measured automatically as in previous studies.^9^^,^^11^ A spectral estimate of the signal to noise ratio was obtained as the ratio between the signal (band within 0.5–40 Hz) and noise (band within 40–100 Hz) power. Signals with signal to noise ratio <13 dB where not included in the analysis.

### Statistical analysis

Continuous variables are represented as mean ± standard deviation if normally distributed and median (25th–75th quantile) if not normally distributed. The Kruskal–Wallis test was used for group comparisons of ARI and RT differences compared with normal voltage. The Student’s *t*-test was used to compare ARI and RT differences transmurally in scar against normal transmural voltage for. Restitution curves were plotted using Lowes regression, and quantile regression used to assess for differences. A *P*-value of <0.05 was considered statistically significant. Analysis was performed using R statistical software.

## Results

### Patient population

Six patients [five male, median age 53 years, interquartile range (IQR) 43–67 years] with VT resistant to medical therapy or recurrent implantable cardioverter-defibrillator shocks, were enrolled in the research study as part of their clinical ablation procedure (*Table 1*). Patient characteristics are shown in *Table 1*. Median LV ejection fraction was 35% (IQR 15–54%).

**Table 1:** Patient characteristics

Study number	Age (years)	LV ejection fraction	Pathology	Medications	Outcome	Scar characteristic
1	44	65	Myocarditis	Amiodarone stopped 6 weeks prior. Bisoprolol	No further VT	Predominant anterior LV epicarial scar and scar boderzone.
2	64	14	Ischaemic cardiomyopathy	Bisoprolol	Further VT recurrence within 6 months	Endocardial and epicardial scar borderzone
3	42	18	Ischaemic cardiomyopathy	Carvedilol	Single shock within 6 months	Predominant apical LV endocardial scar and scar boderzone.
4	68	52	Ischaemic cardiomyopathy	Bisoprolol	No further shocks.	Endocardial and epicardial scar borderzone
5	74	10	Ischaemic cardiomyopathy.	Bisoprolol	No further VT	Endocardial and epicardial scar borderzone
6	34	55	ARVC.	Bisoprolol stopped 5 days before.	Further ICD shocks and further ablation in different region	Endocardial and epicardial scar borderzone

ARVC, arrhythmogenic right ventricular cardiomyopathy; ICD, implantable cardioverter-defibrillator; LV, left ventricle; VT, ventricular tachycardia.

### Example patterns of scar and their association with activation recovery interval and repolarization time

#### Predominant epicardial scar

*Figure 1* demonstrates Endo and Epi voltage maps in a patient with predominant Epi scar (Patient 1). Decapolar catheters were placed across the Epi scar region traversing regions of dense scar, scar borderzone and healthy tissue, and poles were paired for comparison with a transmural geometrically opposed decapolar catheter in the endocardium over normal bipolar voltage (*Figure 1A*). Restitution curves recorded over a region of healthy transmural tissue (*Figure 1B*), demonstrate shorter Epi ARI (median ARI 173 ms vs. 209 ms in endocardium, 38 ms difference at the 50th quantile, *P* < 0.05 across all cycle lengths). Recording in a region of Epi scar borderzone and normal endocardium (*Figure 1C*), demonstrated a smaller non-significant (NS) transmural ARI difference (median Epi ARI 187 ms vs. 207 ms in endocardium, 22 ms difference at 50th quantile *P* = NS across all cycle length). Recording in a region of dense Epi scar and normal Endo tissue (*Figure 1C*), showed prolongation of Epi ARI and no significant transmural ARI difference (median Epi ARI 203 ms vs. 210 ms, 7 ms difference at 50th quantile, *P* = NS across all cycle length). Thus progressive Epi scar resulted in lengthening in ARI, and diminished the transmural ARI gradient.

#### Predominant endocardial scar

**Figure 2:**
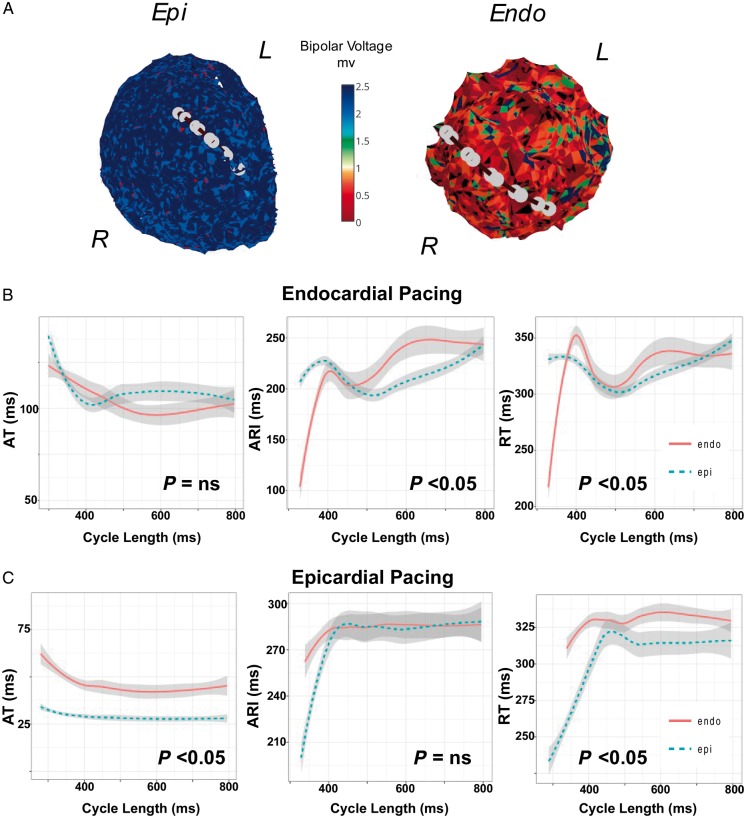
(*A*) Restitution studies in a patient with ischaemic cardiomyopathy and predominant endocardial anterior/apical scar of the left ventricle due to previous myocardial infarction (Patient 3). Catheters position for transmural recordings during restitution studies while pacing (*B*) the Endo and (*C*) Epi are shown. ARI, activation recovery interval; AT, activation time; Endo, endocardium; Epi, epicardium; NS, non-significant; RT, repolarization time.

*Figure 2* shows restitution studies performed across a region of Endo scar in the apex of the LV in a Patient 3. There is extensive low voltage in the Endo anterior wall and apex, with minimal Epi scar. Catheters are opposed transmurally as shown (*Figure 2A*). Restitution studies during Endo (*Figure 2B*) and Epi (*Figure 2C*) pacing are shown.

During Endo pacing, (*Figure 2B*) there is earlier activation of the endocardium (10 ms difference at the 50th quantile, *P* = NS all segments), ARI was longer in the endocardium where there was dense scar (median Endo ARI 226 ms vs. 200 ms Epi, 20 ms difference in the 50th quantile, *P* < 0.05 all cycle lengths), while RT was prolonged endocardially (median Endo RT 320 ms vs. 303 ms Epi, 18 ms difference at 50th quantile, *P* < 0.05 all cycle lengths). Thus despite the earlier Endo AT, Endo RT was delayed due to prolonged ARI in this region.

During Epi pacing, (*Figure 2C*) there was earlier Epi activation (15 ms difference at the 50th quantile, *P* < 0.05 all cycle lengths), ARI was longer in the endocardium than the epicardium (median Endo ARI 291 ms vs. 258 ms epicardially, 33 ms at 50th quantile, *P* < 0.05, but NS across all cycle lengths), and RT longer in the endocardium than the endocardium (median Endo RT 336 vs. 270 in epicardium, 42 ms transmural difference at 50th quartile, *P* < 0.05 all cycle lengths). Thus longer ARI in the scar region of the endocardium resulted in longer Endo RT.

#### Transmural scar

**Figure 3:**
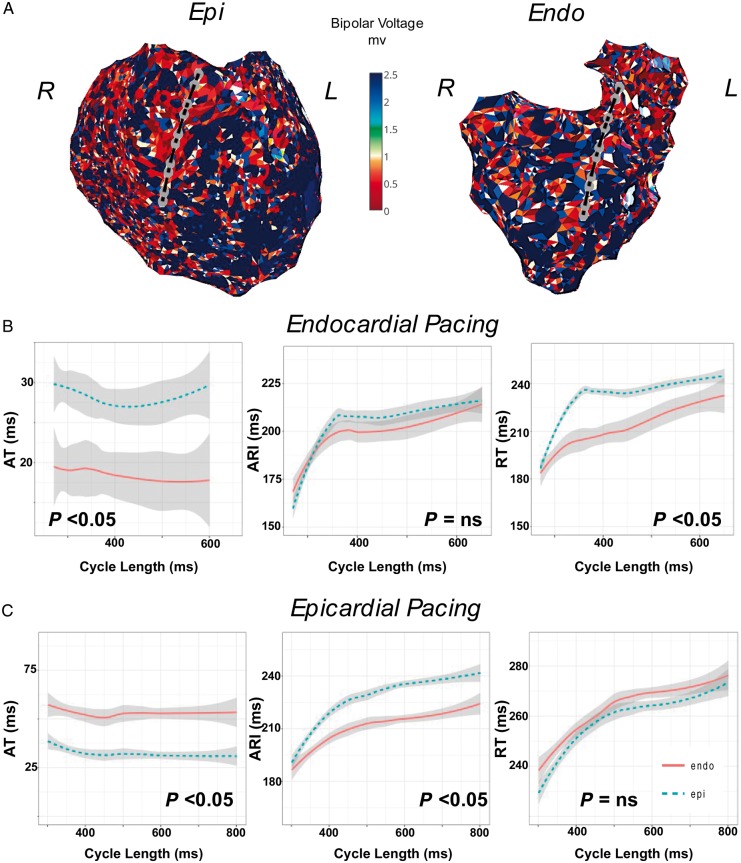
(*A*) Restitution studies in a patient with ARVC across a region of transmural scar in the RVOT (Patient 6). Catheters are positioned transmurally in the anterior RVOT during restitution studies while pacing (*B*) the Endo and (*C*) Epi. ARI, activation recovery interval; ARVC, arrhythmogenic right ventricular cardiomyopathy; AT, activation time; Endo, endocardium; Epi, epicardium; NS, non-significant; RT, repolarization time; RVOT, right ventricular outflow tract.

*Figure 3* shows an example of restitution studies in an arrhythmogenic right ventricular cardiomyopathy patient transmural scar across the right ventricular outflow tract (RVOT). Catheters are transmurally opposed in a region of Endo and Epi scar (*Figure 3A*) and restitution studies during Endo (*Figure 3B*) and Epi (*Figure 3C*) pacing were performed.

During Endo pacing (*Figure 3B*) there is earlier activation of the endocardium (7 ms difference at the 50th quantile, *P* < 0.05 all cycle lengths), however, Epi ARI was longer than in the endocardium (median Epi ARI 209 ms vs. 199 ms Endo, 9 ms at 50th quantile, *P* = NS all cycle lengths), and transmural RT was longer in the epicardium compared with endocardium (median Epi RT 229 ms vs. 218 ms endocardium, transmural RT difference 50 ms at 50th quantile, *P* < 0.05 for all cycle lengths).

During Epi pacing (*Figure 3C*), there was earlier activation of the epicardium (20 ms difference at 50th quantile, *P* < 0.05 all cycle lengths), ARI was again prolonged in the epicardium (median Epi ARI 230 ms vs. 208 ms in epicardium, 20 ms difference at the 50th quantile *P* < 0.05 for all cycle lengths), with no significant transmural RT difference (median Epi RT 260 ms vs. 265 ms in endocardium, 6 ms difference in the 50th quantile, *P* = NS all cycle lengths). Thus ARI in this patient with transmural scar was prolonged in the endocardium and epicardium, with a greater prolongation in the epicardium. Significant differences in transmural RT were seen only during Endo pacing, due to transmural conduction delay.

### Activation recovery interval differences dependent on scar properties

**Figure 4:**
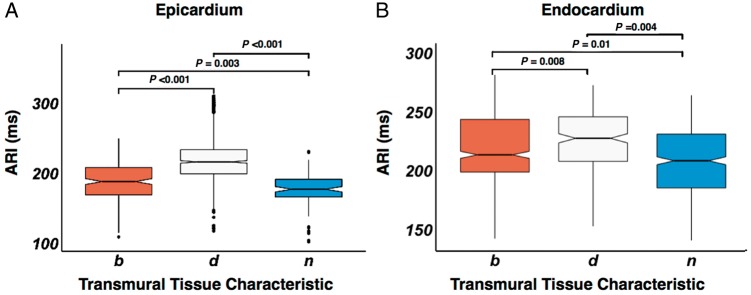
Box and whisker plot of epicardial (*A*) and endocardial (*B*) ARI in relation to the tissue voltage characteristic. Tissue characteristic is denoted on the *x*-axis. Statistical significance between box plots is shown above the plots. ARI, activation recovery interval; b, scar borderzone; d, dense scar; n, normal tissue.

*Figure 4* shows the ARI of endocardium and epicardium in regions of normal voltage, scar borderzone, and dense scar, for all patients at all cycle lengths of restitution pacing. Median Epi ARI (*Figure 4A*) was 173 ms in normal tissue vs. 187 ms at scar borderzone (*P* = 0.003) and 210 ms in dense scar (*P* < 0.001). Median Endo ARI (*Figure 4B*) was 210 ms in normal tissue vs. 222 ms in the scar borderzone (*P* = 0.01) and 238 ms in dense scar (*P* = 0.004). Endocardial scar resulted in an increase in Endo ARI compared with normal tissue but the difference was less than in Epi scar (*Figure 4B*).

### Transmural activation recovery interval and repolarization time gradients are dependent on scar properties

**Figure 5:**
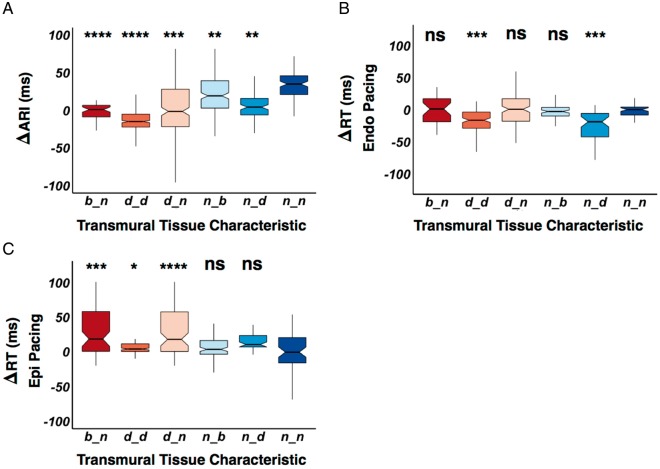
(*A*) Box and whisker plot displaying paired endocardial and epicardial scar pattern, and their association to endocardial subtracted by epicardial ARI (ΔARI), endocardial subtracted by epicardial RT (ΔRT) during endocardial pacing (*B*) and epicardial pacing (*C*). Scar tissue characteristic is denoted on the *x*-axis, the first letter is the characteristic of the endocardium and the second letter is the characteristic of the epicardium. Statistical significance is assessed as a comparison against normal endocardial and epicardial tissue (n_n), with statistical significance denoted above the boxplot. *P < 0.05, **P < 0.01, ***P < 0.001, ****P < 0.0001. ARI, activation recovery interval; b, scar borderzone; d, dense scar; n, normal tissue; NS, non-significant; RT, repolarization time.

**Figure 6:**
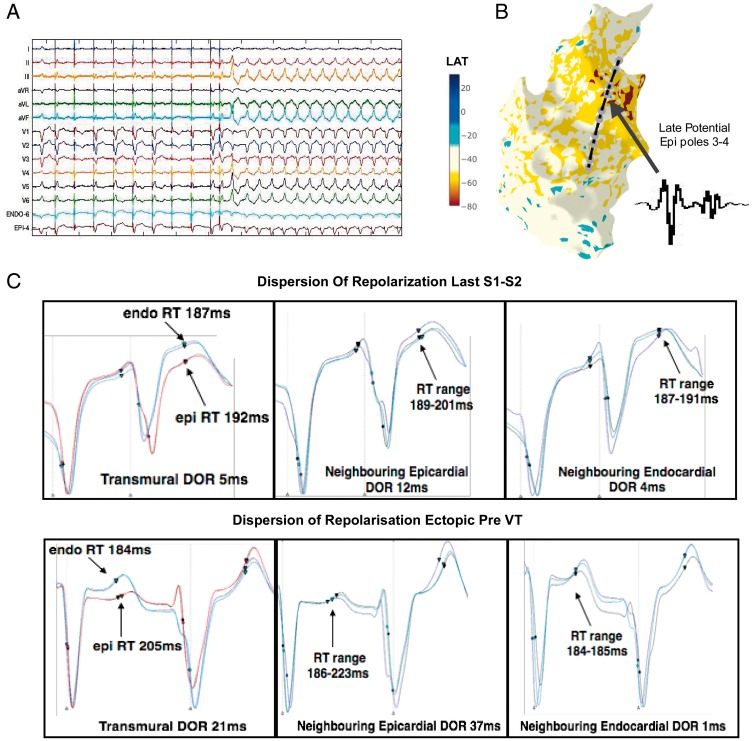
DOR and initiation of VT in an example patient (Patient 6). (*A*) S1–S2 restitution protocol, showing 12-lead ECG and sample unipolar electrograms from the RV endocardium and RV epicardium. Following a train of S1 pacing, an S2 beat is delivered, following this an RV ectopic beat triggers sustained VT. (*B*) RV Endo geometry and activation mapping of the sustained VT showing earliest activation (red area), in the RVOT, along with location of transmurally opposed Endo and Epi decapolar catheters from the restitution study. Poles 3 and 4 of Epi catheter were located over a region of late potentials in sinus rhythm (inset). (*C*) DOR at Epi catheter pole location 3–4 (where fractionation was recorded), in the neighbouring Epi poles to 3–4, and in the neighbouring adjacent linear Endo poles. DOR is shown for the last S2 beat (above panel) and for the ectopic beat subsequent to this which initiates sustained VT. Circles represent activation time, triangles represent repolarization time. DOR, dispersion of repolarization; Endo, endocardial; Epi, epicardial; RV, right ventricular; RVOT, right ventricular outflow tract; VT, ventricular tachycardia.

*Figure 5* shows transmural ARI and RT gradients of all patients in relation to transmural scar properties. Geometrically opposed catheter poles were paired, and their Endo ARI (*Figure 6A*) or RT (Figure *6**B *and *C*) subtracted from the Epi ARI or RT, respectively (**Δ**ARI/RT), at different cycle lengths. Repolarization time is separated for Endo and Epi pacing because activation pattern influences total RT.

In regions of both dense Endo and Epi scar (d_d), there was a significant reduction in **Δ**ARI (*Figure 5A*) due to lengthening of Epi ARI as described in *Figure 3*. Also in regions of dense Endo scar but normal Epi tissue (d_n), Endo borderzone and normal Epi tissue (b_n), and dense Epi scar but normal Endo tissue (n_d) again there was a reduction in **Δ**ARI compared with normal. In normal Endo tissue and scar borderzone epicardially, a significantly reduced **Δ**ARI difference was observed due increase in Epi ARI. Thus regions of scar reduced transmural **Δ**ARI, due to ARI lengthening.

During Endo pacing, regions of dense transmural scar (d_d) and regions of normal Endo tissue with dense scar epicardially (n_d) display negative ΔRT compared with normal tissue, due to the effect of conduction delay and prolonged Epi ARI increasing Epi RT (*Figure 5B*). During Epi pacing (*Figure 5C*), ΔRT was increased compared with normal tissue in d_d, b_n, and d_n tissue due to activation delay and prolonged ARI where there was Endo scar (*Figure 6C*).

### Dispersion of repolarization and arrhythmogenesis

*Figure 6* shows an example where VT was induced in Patient 6 during the S1–S2 restitution protocol. Catheters were transmurally opposed across the RVOT in a region of transmural scar (*Figure 3*). At an S2 interval of 280 ms, the last paced beat is followed by a right ventricular ectopic which initiates VT (*Figure 6A*). Activation mapping showed earliest activation at a site in the anterior RVOT and the position of the decapolar restitution catheters in relation to the activation map is shown (*Figure 6B*). Epicardial poles 3–4, displayed late potentials during sinus rhythm in this region (inset electrograms).

*Figure 6C* shows the transmural unipolar electrograms from the geometrically opposed catheters at the site of earliest activation, as well as the neighbouring electrograms from adjacent electrodes during the last S1–S2 beat and the ectopic beat initiates VT. Transmural dispersion of repolarization (DOR) is 5 ms during the S2 beat, neighbouring Epi DOR is 12 ms in the region where fractionation was recorded and 4 ms in the opposing Endo region. The subsequent ectopic beat increases transmural DOR to 21 ms, and neighbouring Epi DOR to 37 ms, while Endo DOR is 1 ms. This initiates VT, which was mapped to this region of repolarization dispersion and ablation in this region rendered VT non-inducible.

## Discussion

This is the first human study to examine dynamic transmural repolarization changes in VT patients. The main findings are (i) areas of myocardial scar, regardless of pathology, have prolonged ARI compared with normal voltage tissue, (ii) the presence of scar decreases the ARI transmural gradient, the extent of which is dependent on the Endo and Epi scar characteristics, and (iii) alterations in transmural ARI gradient result in localized transmural DOR, dependent on the scar pattern and the activation sequence, which may play an important role in arrhythmogenesis.

### Myocardial scar results in prolongation of action potential duration

Studies of myocardial infarction in dogs^12^ have demonstrated APD shortening acutely, with lengthening as the infarct became chronic. Studies of chronic heart failure and ischaemia have demonstrated prolongation of regional APD.^4^ Our findings are consistent with previous studies showing a prolongation of ARI both in the epicardium and the endocardium in a series of intact human ventricles with a variety of pathologies. We also demonstrate that ARI prolongation is unique to myocardial scar and not a globalized phenomenon as previously reported,^4^ because within patients with diseased ventricles, regions of normal myocardial voltage displayed an ARI consistent to what we have previously demonstrated.^9^ The mechanism of ARI prolongation in scarred tissue may involve a down-regulation of Cx43,^4^^,^^13^ or up-regulation of Ica^14^ which may be an adaptive phenomenon to cause calcium overloading of the cell in order to improve contractility. Down-regulation of Iks and an increase in the KCNQ1(Ikr)-to-KCNE1(Iks) mRNA ratio^15^^,^^16^ have also been reported, and may help counteract down-regulation of Ito epicardially.^14^

### Myocardial scar reduces transmural action potential duration gradients

We have previously demonstrated a transmural gradient in ARI, with longer Endo ARI compared with Epi ARI^9^ in the intact normal human ventricle. In this study, a significant transmural ΔARI was still present in regions of normal Endo and Epi voltage, however, where there was Endo, Epi, or transmural scar/scar-borderzone, ΔARI was diminished(*Figure 5A*). This is consistent with experimental work in heart failure^4^ and in intact human hearts.^5^ The reduction of transmural ΔARI, was related to the scar characteristics, with regions of dense scar both endocardially and epicardially displaying zero to negative ΔARI (*Figure 5A*), due to the greater proportional lengthening of ARI in the epicardium (*Figure 4*). Regions of dense scar in either endocardium or epicardium with normal tissue opposite showed the next lowest reduction in ΔARI; followed by regions where there was scar borderzone opposite normal tissue, where the transmural ARI gradient was similar to normal voltage tissue. This demonstrates that the process of both ARI lengthening and also reduction in ΔARI is a spectrum, dependent on the level of scar and adaptive processes within the scar. Progressive reduction in transmural ΔARI may be an important adaptive process that negates the changes in conduction velocity within the tissue to prevent DOR.

### Alterations in transmural action potential duration may increase dispersion of repolarization and contribute to arrhythmogenesis

The interplay between activation and ARI to influence transmural DOR is demonstrated in Figure *5**B and C*. In regions of dense scar on the endocardium and epicardium, Epi ARI was prolonged to a greater extent, resulting in a negative transmural ΔRT during Endo pacing, due to conduction delay and the prolonged ARI epicardially (*Figure 5B*), and a positive ΔRT during Epi pacing (*Figure 5C*). Where predominant Endo or Epi scar was demonstrated, due to prolongation of ARI in the scar region transmural DOR occurred when pacing on the side opposite the scar (*Figure **5**B *and *C*) due to the addition of conduction delay and the prolonged ARI.

Although DOR is only one factor in the initiation of arrhythmia, we demonstrate an example of DOR in a region of VT initiation in a patient with scar present both endocardially, and epicardially across the RVOT (*Figure 6*). In this patient due to the greater lengthening of Epi ARI, Epi RT was longer during Endo pacing (*Figure 3*), with negative ΔARI and ΔRT (*Figure**5**A* and *B*). When VT was initiated by an ectopic (*Figure 6*) there was significant transmural and neighbouring Epi DOR along the site of early activation, with relatively little Endo DOR. The threshold for local transmural DOR seen here would be predicted to promote local functional re-entry.^17^

Additionally, we mapped earliest VT activation, within a region of prolonged repolarization and fractionated late potentials (*Figure 6B*) during sinus rhythm. Late potential mapping is an important tool in substrate ablation for patients with VT,^18^ however, the mechanism of these potentials is unclear. Canine studies have suggested fractionation occurs over regions of myocardial disarray and conduction slowing,^12^ while isolated tissue studies have suggested fractionation may occur in regions of localized Phase 2 re-entry^19^ or DOR.^20^ Our findings suggest that abnormal DOR plays an important role the mechanism of fractionated late potentials, and is the first demonstration of this in the intact human heart.

### Limitations

Data were confined to multi-electrode sequential contact mapping, without single beat global assessment of repolarization as may be provided by non-contact methods. Additionally accepted voltage cut-offs for scar threshold were applied based on published data,^1^^,^^2^ however, debate exists as to what the correct optimal voltage cut-offs should be. Nevertheless, we were able to record APD differences in these regions based on the voltage criteria used. Finally, stimulus site usually affects APD closer to the capture site as we have previously demonstrated in the structurally normal heart.^9^ We did not demonstrate such changes perhaps due to the limited number of patients studied and the varied APD properties of the scar.

## Conclusions

This is the first human study to demonstrate that regions of electrical scar within the myocardium have prolonged ARI. We demonstrate that ARI prolongation is related to scar pattern and influences DOR depending on the activation sequence. This has important implications when constructing clinically relevant computational models or designing novel therapeutic interventions. Additionally, we demonstrate an example of DOR at a site of fractionation and earliest VT activation. This may provide further insights into the mechanisms of VT initiation, but also new methods to better delineate the pro-arrhythmic substrate within the ventricle by rapidly mapping dynamic differences in ARI and RT as part of routine 3D substrate mapping.

## Funding

This work was supported by University College London Hospitals Biomedicine National Institute for Health Research. N.T.S. was supported by a British Heart Foundation Clinical Research Training Fellowship [FS/14/9/30407]. M.O. was supported by Marie Curie Fellowship [IEF-2013]. P.D.L. and P.T. were supported by the Medical Research Council [G0901819].

**Conflict of interest:** none declared.
